# Alterations in metabolome and microbiome: new clues on cathelicidin-related antimicrobial peptide alleviates acute ulcerative colitis

**DOI:** 10.3389/fmicb.2024.1306068

**Published:** 2024-02-06

**Authors:** Nan Jiang, Zhongyuan Liu, Haiyang Wang, Lichun Zhang, Mengjiao Li, Gaoqian Li, Chang Li, Bo Wang, Cuiqing Zhao, Liming Liu

**Affiliations:** ^1^Department of Trauma Center, China-Japan Union Hospital of Jilin University, Changchun, Jilin, China; ^2^College of Animal Science and Technology, Jilin Agricultural Science and Technology University, Jilin, Jilin, China; ^3^Department of Trauma Center and Gastrointestinal Surgery, China-Japan Union Hospital of Jilin University, Jilin, China

**Keywords:** cathelicidin related antimicrobial peptide (cramp), colitis, inflammation, intestinal barrier, metabolome, microbiome

## Abstract

Ulcerative colitis (UC) is a chronic and recurrent inflammatory disease of the gastrointestinal tract. This study aimed to determine the effect of cathelicidin-related antimicrobial peptide (Cramp) on dextran sulfate sodium (DSS)-induced acute experimental colitis in mice and to investigate the underlying mechanisms. Acute UC was induced in C57BL/6 mice with 3% DSS for 7 days, 4 mg/kg b.w. synthetic Cramp peptide was administrated once daily starting on day 4 of the experimental period. Mice were evaluated for body weight, colon length, colon histopathology, and inflammatory cytokines in colon tissue. Using 16 s rRNA sequencing, the composition structure of gut microbiota was characterized. Metabolomic profiling of the serum was performed. The results showed that DSS treatment significantly induced intestinal damage as reflected by disease activity index, histopathological features, and colon length, while Cramp treatment significantly prevented these trends. Meanwhile, Cramp treatment decreased the levels of inflammatory cytokines in both serum and colonic tissue on DSS-induced colitis. It was also observed that DSS damaged the integrity of the intestinal epithelial barrier, whereas Cramp also played a protective role by attenuating these deteriorated effects. Furthermore, Cramp treatment reversed the oxidative stress by increasing the antioxidant enzymes of GSH-PX and decreasing the oxidant content of MDA. Notably, compared to the DSS group, Cramp treatment significantly elevated the abundance of Verrucomicrobiota at the phylum level. Furthermore, at the genus level, Parasutterella and Mucispirllum abundance was increased significantly in response to Cramp treatment, although Roseburia and Enterorhabdus reduced remarkably. Metabolic pathway analysis of serum metabolomics showed that Cramp intervention can regulate various metabolic pathways such as α-linolenic acid, taurine and hypotaurine, sphingolipid, and arachidonic acid metabolism. The study concluded that Cramp significantly ameliorated DSS-induced colonic injury, colonic inflammation, and intestinal barrier dysfunction in mice. The underlying mechanism is closely related to the metabolic alterations derived from gut microbiota.

## 1 Introduction

Inflammatory Bowel Disease (IBD) is a group of disorders causing chronic inflammation disorders of the intestinal tract, including Crohn’s disease (CD) and ulcerative colitis (UC), in which patients suffer from pain, vomiting, diarrhea, and other symptoms (Ananthakrishnan, 2015). In recent years, the incidence of IBD has developed into a global disease (Kaplan and Ng, 2017), which is caused by multiple interactions of genetic, microbial, and immunological factors. Although the specific underlying mechanism of IBD is still not clear at present (Kaplan and Ng, 2016), but commonly associated with aberrant immune responses, excessive oxidative stress, and unbalanced gut microbiota (Li et al., 2023). The gastrointestinal tract is the largest, highly complex and dynamic micro-ecosystem in the human, consisting of various types of epithelial cells and commensal microorganisms (Bengmark, 1998). Studies suggests that the intestinal structure and luminal environment work together to maintain intestinal homeostasis. Disruption of this balance is thought to play a role in the pathogenesis of IBD (Chang, 2020). Antimicrobial peptides (AMPs) are a diverse group of biologically active compounds that play a key role in host defense and maintenance of tolerance to commensal microorganisms (Cunliffe and Mahida, 2004; Chung and Raffatellu, 2019).

Mouse cathelin-related antimicrobial peptide (Cramp) and its homologue human cathelicidin (LL-37), as one of the AMPs, plays an important role in intestinal microbe ecosystems (Yoshimura et al., 2018). As reported earlier, Cramp has a wide range of anti-microbial and immunomodulatory functions, and plays a role in maintaining the integrity of the epithelial barrier (Otte et al., 2009; Koon et al., 2011). It has been reported that cathelicidin mediated immune responses in intestinal colitis (Ho et al., 2013). LL-37 expression is elevated in the colonic mucosa of patients with UC but not in those of patients with CD (Schauber et al., 2006). Cramp knockout mice were previously demonstrated to develop more severe colitis in response to DSS than WT mice (Koon et al., 2011), which was associated with more mucosal disruption, higher levels of proinflammatory cytokines production, and increased infiltration of intestinal inflammatory, culminating in decreased mouse survival (Yoshimura et al., 2018). Furthermore, intrarectal administration of exogenous CRAMP attenuates DSS-induced colitis by protecting the mucous layer, decreasing the production of proinflammatory cytokine, and suppressing apoptosis of epithelial (Tai et al., 2007). In addition, the maintenance of epithelial barrier integrity by Cramp is mainly achieved by increasing the expression of tight junction proteins (Marin et al., 2019). The interaction between the gut microbiome and colon mucosa is well-established to cause inflammatory bowel disease and impaired healing. Notably, UC is known to be associated with dysbiosis in the gut microbiota of patients and mice (Imhann et al., 2018; Walujkar et al., 2018; Parada Venegas et al., 2019; Paramsothy et al., 2019), and CRAMP expression may be regulated by gut microbiota and metabolites such as short-chain fatty acids or proteases (Potempa and Pike, 2009; Sun et al., 2015). Furthermore, a study shows that Cramp alters the composition of the gut microbiome in celiac disease mice (Ren et al., 2021). Collectively, these suggest that Cramp attenuates colitis associated with inflammation, intestinal tight junctions, and intestinal microbiome. However, the mechanistic basis of these observations remains unclear.

Studies have shown that IBD can lead to abnormalities in multiple metabolic pathways, including fatty acids, amino acids, and bile acids. The gut microbiota can influence host metabolites and is detected in a wide range of biological tissues. Extensive changes in the fecal, serum, and urinary metabolomes have been documented in IBD, which has provided a means of classifying patients with IBD, as well as insights into putative mechanisms and the discovery of novel associations (Lavelle and Sokol, 2020). In addition, to our knowledge, no studies have investigated the mechanism of Cramp on DSS-induced colitis by metabolomic analysis.

In the present study, we investigated the protective effects of Cramp against DSS-induced colitis in mice and for the first time explored the underlying mechanism from the metabolic perspective. The study demonstrated the protective effect of Cramp on a mouse model of DSS-induced colitis and its mechanism, highlighting the modulatory effects on inflammation and intestinal barrier. In addition, the 16S rRNA sequencing and metabolomics analysis were performed to reveal new insights into the features of cramp applications in colitis treatment.

## 2 Results

### 2.1 Cramp alleviates signs and symptoms of DSS-induced colitis in mice

**Figure 1:**
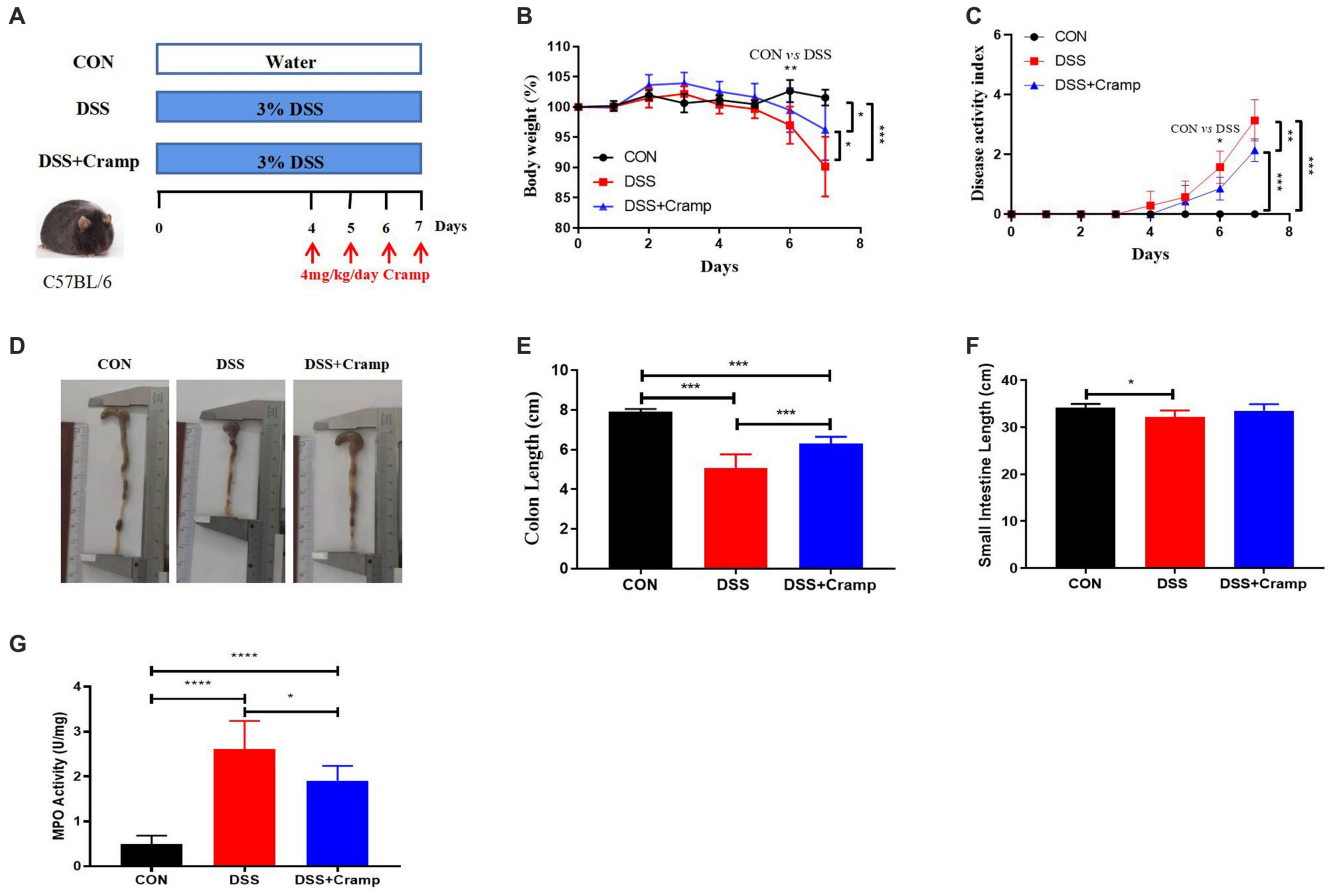
Cramp ameliorated clinical symptoms of DSS-induced colitis in mice. **(A)** Schematic diagram of an experimental design. **(B)** Body weight was recorded daily for three groups of mice. **(C)** Disease activity index (DAI) scores. **(D)** Representative image of the colon. **(E)** Colon length. **(F)** Small intestine length. **(G)** Colon myeloperoxidase (MPO) enzymatic activity.

The effects of Cramp on weight loss, colonic injury, and inflammation were investigated in a DSS-induced mice model of acute colitis. Mice were provided with drinking water containing 3% DSS for 7 days, and synthetic Cramp peptide was injected intraperitoneally at 4 mg/Kg/day/mouse from the fourth day (Figure 1A). Compared with the CON group, mice in the DSS group exhibited significant weight loss (Figure 1B) and increased disease activity index (DAI) score (Figure 1C) starting from the sixth day. Shortened colon (Figures 1D,E) and small intestinal (Figure 1F) lengths were observed in DSS-induced colitis mice at the end of the experiment compared with the CON group. Furthermore, DSS-induced colitis mice exhibited significantly higher myeloperoxidase (MPO) activity in comparison to the CON group (Figure 1G). In contrast, the group treated with Cramp significantly ameliorated DSS-induced colitis, as evidenced by improved weight loss (Figure 1B), DAI score (Figure 1C), colonic shortening (Figures 1D,E), and MPO activity (Figure 1G) compared with the DSS group.

### 2.2 Cramp ameliorates colonic lesions and DSS-induced colitis

**Figure 2:**
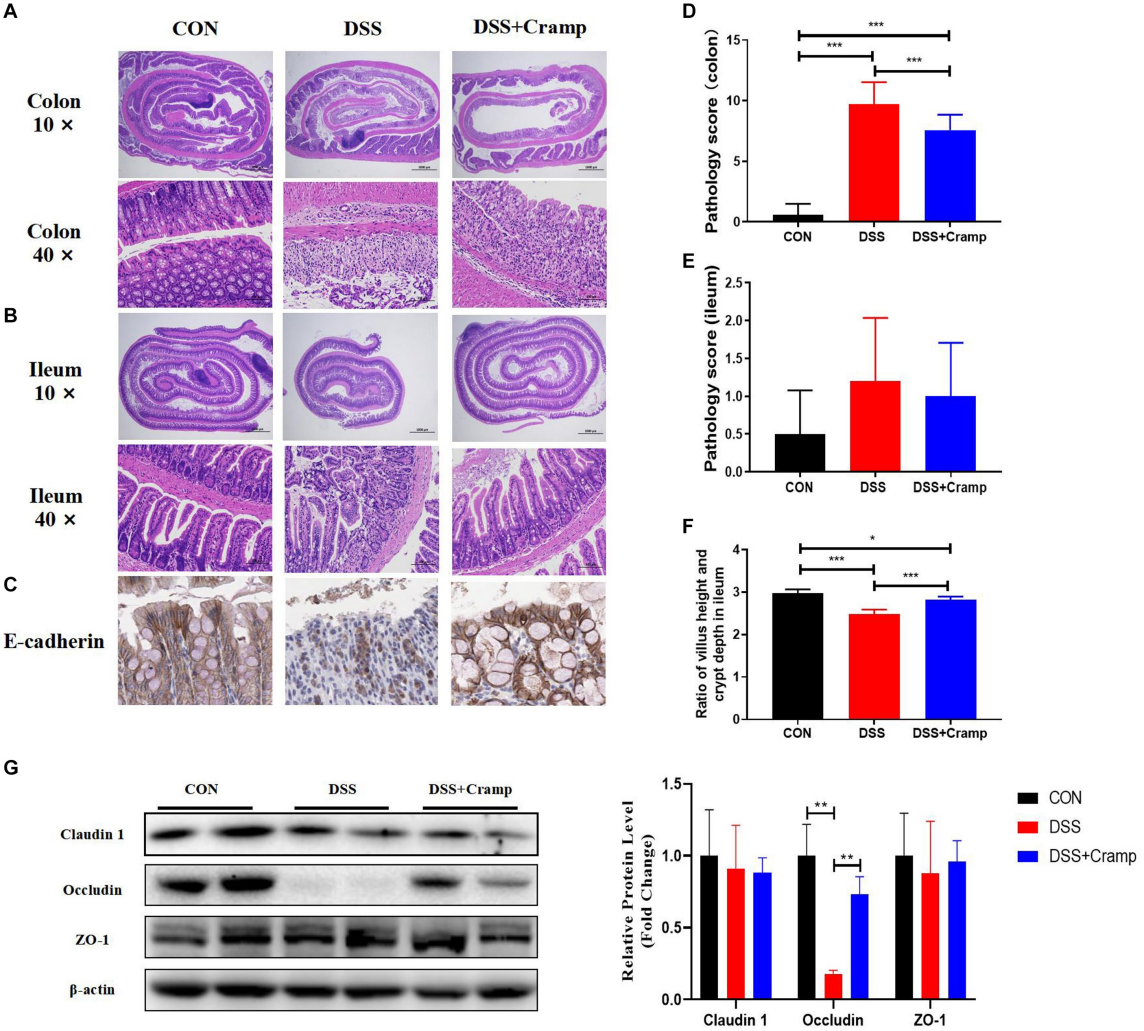
Cramp improved the histopathological injury of DSS-induced colitis in mice. Representative images of colon **(A)** and small intestinal **(B)** damage by H&E staining. **(C)** Representative images of E-cadherin immunohistochemical staining. Colonic **(D)** and small intestinal **(E)** histology score. **(F)** Ileum villus height/crypt depth ratio. **(G)** Immunoblots (left) and quantification (right) of colonic Claudin 1, Occludin, and ZO-1. β-actin served as a loading control.

At the end of the experiment, the entire colon and small intestine of the mice were collected and histologically examined. DSS-induced colitis is characterized by ulceration, epithelial cell death, crypt distortion, and inflammatory cells infiltrating into the lamina propria and submucosa based on histological assessment of the colonic tissue. Cramp treatment significantly improved the severity of colonic mucosal lesions, reduced the infiltration of inflammatory cells into the mucosa and submucosa, preserved the integrity of the colonic mucosa, and reduced the histological score (Figures 2A,D). Cramp had a similar effect on the small intestinal lesions associated with DSS-induced colitis, but there was no difference in pathology score (Figures 2B,E). However, the villi height/crypt depth ratio of ileal was significantly reduced in the DSS group and significantly increased in the DSS + Cramp group (Figure 2F).

Tight junction (TJ) is an important permeable intercellular barrier that plays a key role in the integrity of the intestinal epithelial barrier. In this study, we detected the expression of TJ proteins, such as occludin, ZO-1, and E-cadherin. As shown in Figure 2C, the immunohistochemical of E-cadherin served to confirm that the mucosa was lost in the colonic tissue from the DSS group. Cramp treatment maintained the integrity of the TJ by increasing the expression of E-cadherin. Furthermore, we observed that the expressions of occludin remarkably reduced in the colonic tissues of the DSS group. After Cramp treatment, the reduced expressions of occludin proteins were counteracted (Figure 2G), which provided another strong evidence for the protective effects of Cramp against colitis in mice.

### 2.3 Cramp ameliorates immune regulation in DSS-induced colitis in mice

**Figure 3:**
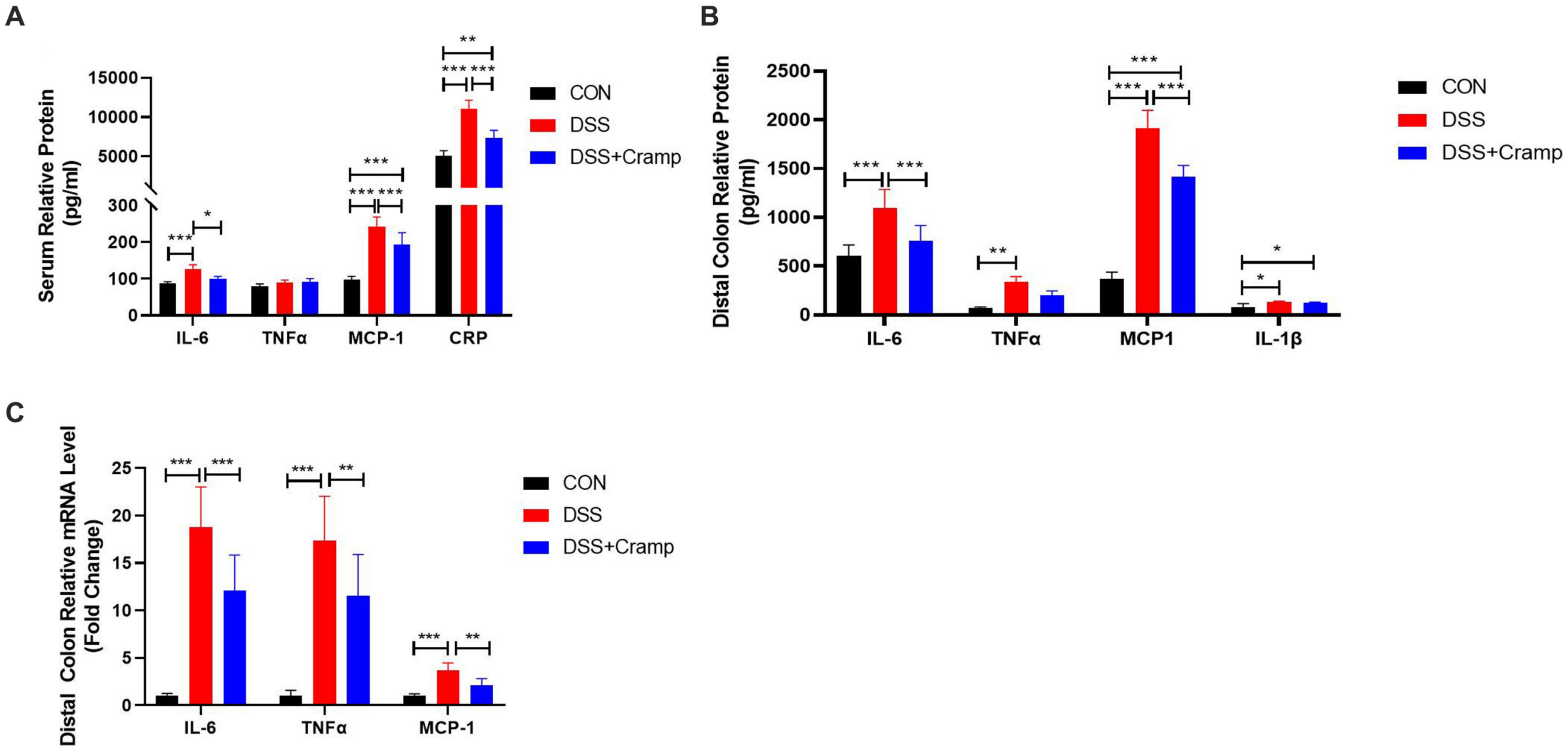
Cramp attenuated inflammation of DSS-induced colitis in mice. **(A)** Serum levels of inflammatory mediators (IL-6, TNFα, MCP-1, and CRP). **(B)** Inflammatory mediator secretion in colon tissue culture (IL-6, TNFα, MCP-1, and IL-1β). **(C)** mRNA expression of inflammatory mediators in colon tissue (IL-6, TNFα, and MCP-1).

Since colitis is a systemic inflammatory disease, in addition to colonic tissues, we also examined serum levels of cytokine and C-reactive protein (CRP). As shown in Figure 3A, the levels of IL-6, MCP-1 and CRP in the serum were significantly increased in the DSS group, while their levels were significantly decreased in the Cramp group. In addition, to evaluate the inflammatory status in colon tissues, we detected the concentrations of inflammatory cytokine in the distal colon by ELISA. The results were shown in Figure 3B, the protein levels of IL-6, TNF-α, MCP-1, and IL-1β were significantly increased in the DSS group compared with those in the CON group. As expected, the levels of IL-6 and MCP-1 were remarkably decreased after Cramp treatment. Furthermore, colonic inflammatory cytokine expression was examined by RT-qPCR. DSS markedly up-regulated the mRNA levels of TNF-α, IL-6, and MCP-1 in colonic tissues, while Cramp remarkably reversed this trend (Figure 3C). These data demonstrated that Cramp reduced the level of inflammatory factors in serum and colonic tissue of DSS-induced colitis.

### 2.4 Cramp relieved oxidative stress in DSS-induced colitis in mice

**Figure 4:**
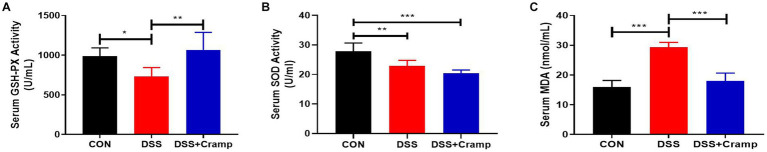
Cramp inhibits oxidative stress of DSS-induced colitis in mice. **(A)** Activity of GSH-PX in the serum. **(B)** Activity of SOD in the serum. **(C)** MDA level in the serum.

As seen in Figure 4, DSS significantly decreased the activities of antioxidants (SOD and GSH-PX), while significantly increasing the content of oxidant (MDA). Conversely, Cramp treatment reversed this effect and led to a significant increase in the activities of GSH-PX (Figure 4A), while significantly decreasing the content of MDA (Figure 4C). These results demonstrate that Cramp intervention alleviates oxidative stress in DSS-induced colitis in mice.

### 2.5 Cramp alters the gut dysbiosis in DSS-induced colitis

**Figure 5:**
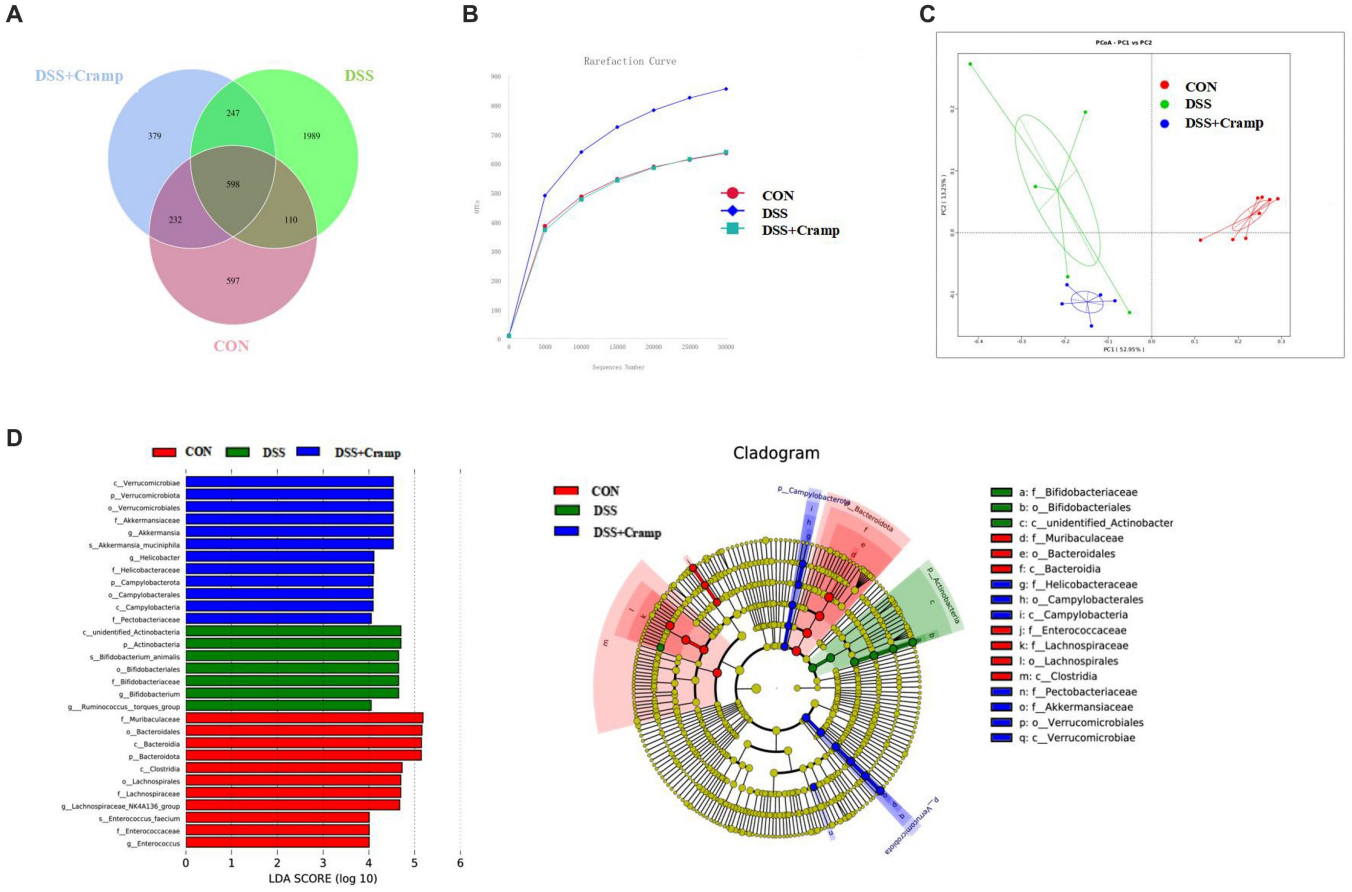
Cramp treatment modulated gut microbiota structure in DSS-induced colitis. **(A)** Venn diagram indicating the differential numbers of OTUs in each group. **(B)** Rarefaction curves of feces samples. **(C)** Principal Co-ordinates Analysis (PCoA) of gut microbiota. **(D)** Linear discriminant analysis (LDA) score in each group (left). Cladogram of gut microbiota in each group (right).

Alterations in the gut microbiota may contribute to the development of IBD. To investigate whether Cramp could reverse DSS-induced dysbiosis, we assessed the composition of the colonic microbiota. The variation in operational taxonomic units (OTUs) of the three groups is depicted in Venn diagrams. A total of 4,162 OTUs were obtained. The Venn diagram shows that 598 OTUs coexisted in all three groups; 708 OTUs coexisted between the CON and DSS group, and 845 OTUs coexisted between the DSS and the DSS + Cramp group. The data showed different diversity of OTUs in each group (Figure 5A). As shown in Figure 5B, the rarefaction curve for each group tended to be flat, indicating that the sequencing results are credible. Principal-coordinate analysis (PCoA) analysis showed separation between CON and DSS groups, which was also observed between the DSS + Cramp and DSS groups (Figure 5C).

In order to discriminate the gut bacterial communities among these three groups, The LEfSe analysis was then used to identify the significant features of microflora associated with Cramp treatment. As shown in Figure 5D, Bacteroidetes were abundant in the CON group. The abundance of Actinobacteria was overrepresented in the DSS group, while the higher abundance of Verrucomicrobiota at the phylum level and enrichment of Akkermansia at the genus level were observed in the DSS + Cramp group.

### 2.6 Cramp affects microbial abundance in DSS-induced colitis

**Figure 6:**
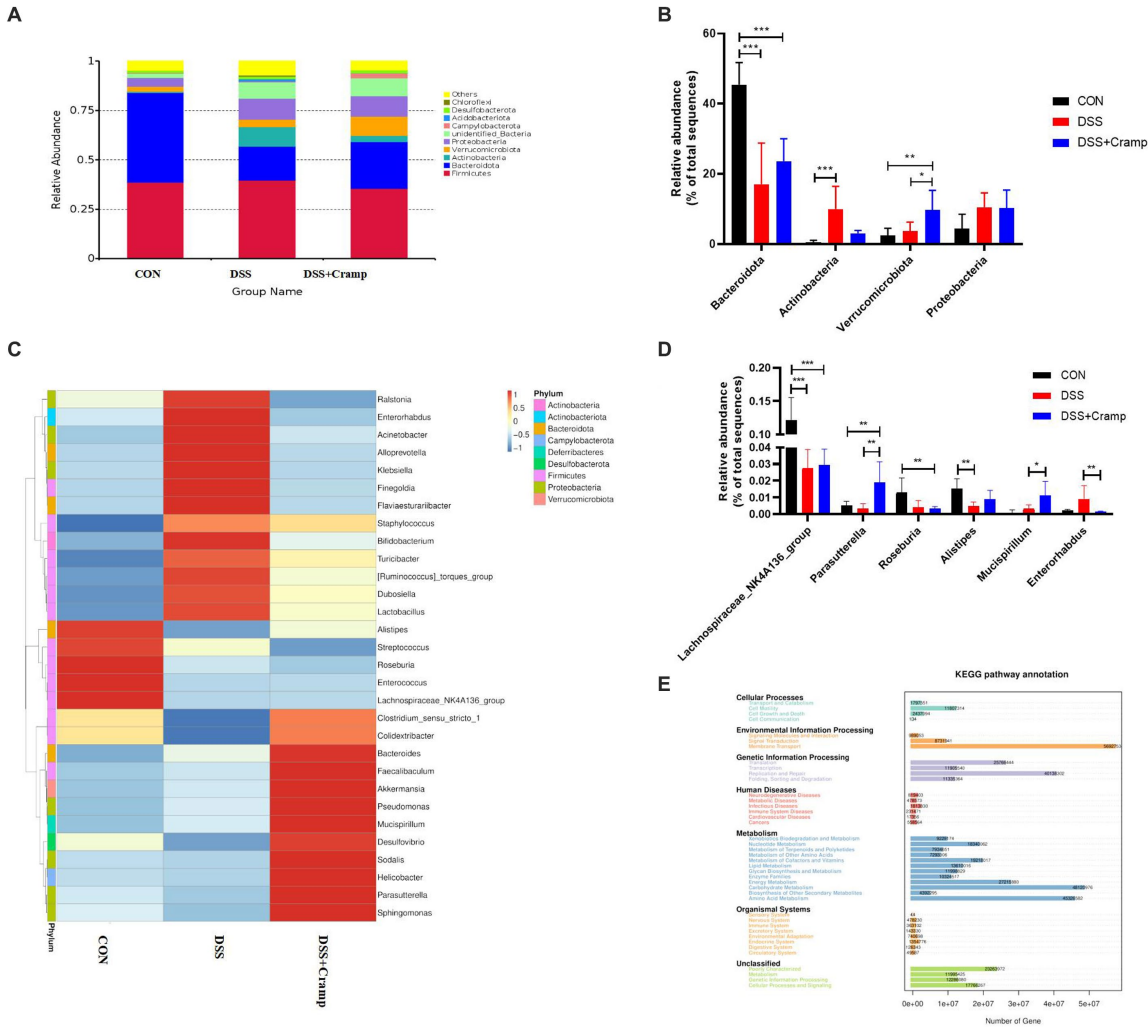
Cramp treatment regulated the gut microbiota composition in DSS-induced colitis. **(A)** Relative abundance of gut microbiota at the phylum level in each group. **(B)** Comparison of the relative abundance at the phylum levels among each group. **(C)** Heatmap of the gut microbiota at the genera level in each group. **(D)** Comparison of the relative abundance at the genus levels among each group. **(E)** PICRUSt predicted analyses.

At the phylum level, the colonic microbiota was dominated by Bacteroidetes, Firmicutes, and Proteobacteria (Figure 6A). Moreover, as shown in Figure 6B, the abundance of Actinobacteria significantly increased, while the abundance of Bacteroidetes significantly decreased in the DSS group. Furthermore, compared to the DSS group, Cramp treatment did not affect the abundance of Actinobacteria and Bacteroidetes. However, Cramp induced a remarkable increase in the abundance of Verrucomicrobiota. The top thirty most abundant microbial genera were chosen for analysis. Lactobacillus, Bifidobacterium, Akkermansia, Lachnospiraceae_NK4A136_group, and Dubosiella were the top five predominant genera (Figure 6C). In addition, DSS significantly suppressed, the relative abundance of Lachnospiraceae_NK4A136_group and Alistipes in the colon. However, Cramp was unable to restore the dysbiosis of these genera. It is worth noting that Parasutterella and Mucispirllum abundance was increased significantly in response to Cramp treatment, although Roseburia and Enterorhabdus reduced remarkably (Figure 6D).

We then used the PICRUSt analysis and the KEGG pathway orthology to investigate the effect of Cramp on potential metabolic pathways in the gut microbiota of colitis mice. As shown in Figure 6E, several pathways of the gut microbiome changed significantly among the three groups, especially the pathways of amino acid metabolism, carbohydrate metabolism, energy metabolism, membrane transport, and replication and repair, respectively.

### 2.7 Serum metabolome profile and biomarker annotation

**Figure 7:**
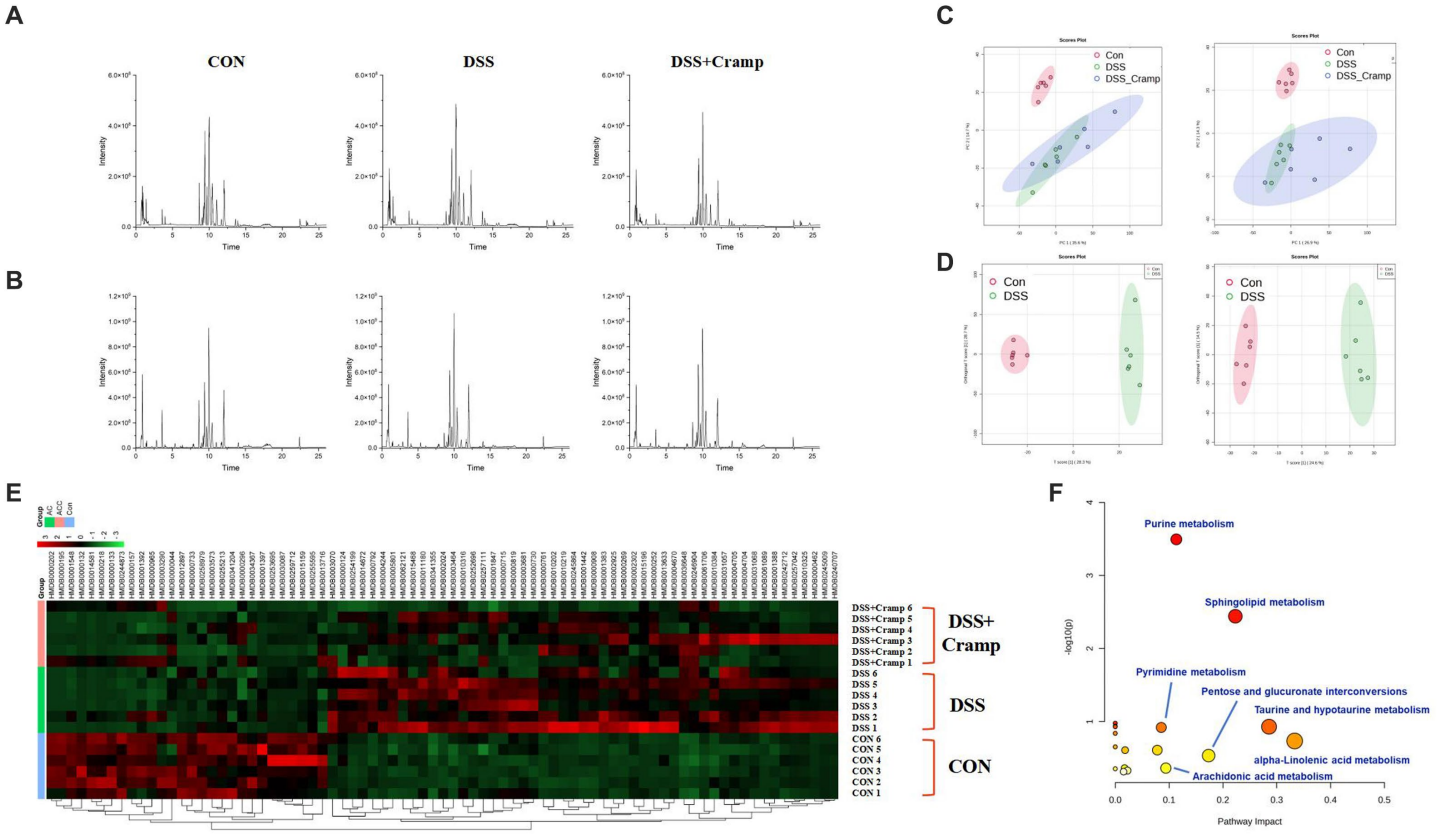
Effects of Cramp on the serum metabonomic profiling by LC–MS. Representative base peak chromatograms of serum samples acquired from organic extracts [**(A)** ESI+ and **(B)** ESI−]. **(C)** PCA score plots of the CON group, DSS group, and DSS + Cramp group based on the data acquired from 50% methanol in water extracts (left, ESI+, and right, ESI-). **(D)** OPLS-DA score plots/Volcano plots/S-plot of CON group and DSS group based on the data acquired from 50% methanol in water extracts (left, ESI+, and right, ESI−). **(E)** Heatmap of the changes in the intensities of biomarkers and bubble plot **(B)** of the metabolic pathway analysis. **(F)** The pathway analysis is visualized by a bubbles plot (the color represents the *p* value, and the darker the color, the more significant it is; the size of the circle represents the percentage of differential metabolites in the enrichment results, and the larger the number, the larger the circle).

To discover the potential metabolites and pathways induced by Cramp, an untargeted metabolomic analysis of serum was performed using UHPLC-Q-Orbitrap/MS. Figures 7A,B demonstrates the base peak chromatograms of the different groups. The peaks differed significantly in terms of retention time and peak intensity. However, because each chromatogram contains many ions, identification of metabolites requires multivariate statistical analysis. After data processing using MS-Dial, a scoring plot was created based on the LC–MS data of the serum extracts in both positive and negative ion modes as shown in Figure 7C. We can observe that there is a clear distinction between the CON group and the DSS group. After Cramp treatment, although the DSS + Cramp group still partially crossed over from the DSS group, there was a trend toward separation between the two groups, suggesting that Cramp treatment could partially restore the metabolic changes in the DSS group.

As shown in Figure 7D, VIP > 1 characterized markers were selected for screening to specify the serum metabolites that contributed most to the separation of samples from the CON and DSS groups. These biomarkers were identified through a database search using precision mass spectrometry and tandem mass spectrometry information. A total of 79 ions were identified based on the above procedure and are listed in Supplementary Table S1.

### 2.8 Metabolic pathway analysis

The results of serum differential metabolites are shown in Supplementary Table S1, where a total of 79 biomarkers were identified between the CON and DSS groups. Among the 79 differential metabolites, DSS-induced colitis upregulated 51 metabolites and downregulated 28 metabolites. These differential metabolites were plotted with a heatmap as shown in Figure 7E, most of the DSS-induced changes in metabolite intensities were negatively regulated after Cramp treatment. Pathway analysis of the identified biomarkers resulted in multiple metabolic pathways as shown in Figure 7F, including alpha-linolenic acid (ALA) metabolism, taurine and hypotaurine metabolism, sphingolipid metabolism, arachidonic acid (AA) metabolism, etc. This suggests that Cramp might mitigate DSS-induced metabolic changes by modulating these pathways.

### 2.9 Correlations between serum metabolome profile and gut bacteria

**Figure 8:**
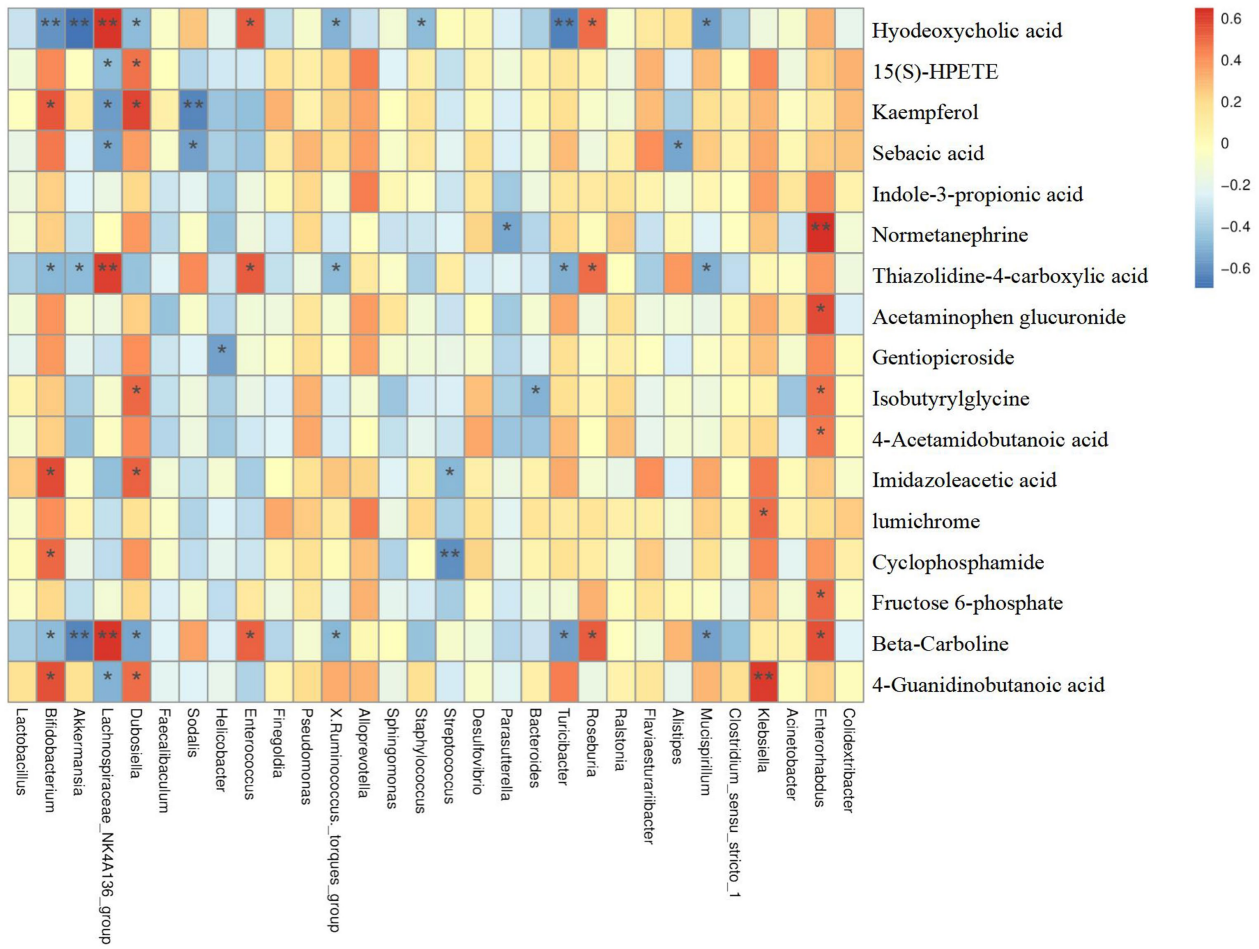
Correlation analysis among differential serum metabolites and gut microbiota.

As mentioned previously, we putatively identified a total of 79 differential metabolites between the CON and DSS groups. Notably, treatment of Cramp significantly improved 18 metabolites when compared to the DSS group. To explore the relationship between serum metabolome and gut microbiome, a correlation analysis was performed between differential serum metabolite levels and gut microbiota at the genus and OTU levels. Results showed in Figure 8, Parasutterella was negatively correlated with Normetanephrine, Mucispirllum was negatively correlated with sebacic acid, thiazolidine-4-carboxylic acid, and beta-Carboline, while Roseburia was positively correlated with hyodeoxycholic acid, thiazolidine-4-carboxylic acid, and beta-Carboline.

## 3 Discussion

IBD is a complex inflammatory disorder of the colon that affects millions of people worldwide (Kaplan, 2015). However, its pathogenesis is still unclear. Recent studies have shown that the possible mechanisms of UC, one of the typical inflammatory gastrointestinal diseases, include inflammatory responses, structure disorder of the intestinal microbiome, and intestinal barrier dysfunction (Wlodarska et al., 2015; Britton et al., 2019). It has been shown that Cramp expression was increased in the DSS-induced colitis mice model and that genetic deletion of Cramp in mice resulted in more severe forms of DSS-induced colitis (Koon et al., 2011). Fabisiak et al. (2021) showed that intraperitoneal injection of Cramp reduced ulcerations and macroscopic scores in both DSS and TNBS-induced colitis models. In the present study, the 3% DSS-induced mice model of colitis was consistent with the pathologic changes and clinical signs of UC patients. As expected, we found Cramp treatment significantly improved the clinical manifestations of UC, such as diarrhea, bloody stool, and weight loss. Various studies have confirmed the role of AMPs in UC, with potential involvement via regulating intestinal barrier function (Shang et al., 2021), upregulation of anti-inflammatory factors, and downregulation of pro-inflammatory factors. It has also been shown that higher serum LL-37 is associated with a reduced risk of inflammation and clinical recurrence in patients with colitis (Gubatan et al., 2020). In contrast, Cramp treatment significantly reduced DSS-induced elevated colonic inflammatory cytokine levels in experimental colitis (Huynh et al., 2019). Our results suggested Cramp treatment has a significant protective effect against anti-inflammatory effects in UC mice. These findings suggest a beneficial effect of Cramp in the treatment of UC and encouraged us to further explore the potential mechanisms by which Cramp alleviates UC.

The intestinal epithelium forms a physical barrier that regulates microbial colonization and prevents them from infiltrating the epithelium (Allaire et al., 2018). Disruption of the intestinal barrier has been reported to be a marker of intestinal inflammation and contributes to the pathogenesis of IBD (Zhao et al., 2020; Salimi et al., 2023). In our study, the expression of gut barrier-related genes was evaluated. As shown in Figure 2G, DSS significantly reduced the protein expression levels of E-cadherin and Occludin, while the effects of Cramp were reversed to varying degrees. Previous study has reported the effect of Cramp treatment on the intestinal barrier. Similar to the present study, Ren et al. reported that Cramp increased the expression of ZO-1, ZO-2, claudin-1, and occludin, thereby counteracting gluten-induced gut barrier dysfunction (Ren et al., 2021). The above-mentioned results indicated that DSS disrupts the integrity of the intestinal epithelial barrier and that Cramp exerts a protective effect by reversing these trends. A significant increase in oxidative stress is associated with diminished colonic mucosal barrier function due to a sharp decline in tight junction proteins (Seo et al., 2014). Indeed, an imbalance of redox homeostasis is a crucial inducing factor in the development and progression of IBD, causing oxidative stress in the gastrointestinal tract through increased production of reactive oxygen species (ROS) (Tian et al., 2017). Our previous findings indicate that Cramp knockout mice have increased hepatic ROS production, which revealed a possible mechanism of Cramp alleviating DSS-induced colitis from an antioxidant perspective (Li et al., 2020). In this study, the DSS treatment significantly increased the MDA contents in the serum, a biomarker of oxidant. However, Cramp treatment reduced the MDA levels (Figure 4C). Moreover, the intracellular enzymatic antioxidants of GSH-PX and SOD in the serum of DSS group were highly decreased compared with the CON group (Figures 4A,B). The treatment of Cramp significantly increased the GSH-PX activity, indicating that the Cramp could enhance antioxidant ability.

Gut ecology consists of host intestinal tissues, gut microbiota and their derived metabolites, all of which contribute to be maintenance of a balanced intestinal environment. Increasing evidence strongly suggests that IBD is caused by an inflammatory response to abnormal changes in gut microbiota (Elzayat et al., 2023). Research has reported that the abundance of Proteobacteria is increased in the feces of IBD patients, whereas members of the Bacteroides phylum are decreased (Hourigan et al., 2015; Matsuoka and Kanai, 2015). The changes in the gut microbiota composition and function were observed after Cramp treatment of IBD (Pound et al., 2015). Paradoxically, intrarectal Cramp administration did not alter the intestinal microbial imbalance although it reduced the severity of DSS-induced colitis in mice (Gubatan et al., 2020). Therefore, to further explore the potential mechanism of intraperitoneal injection of Cramp for the treatment of UC, in this study, 16S rRNA sequencing was used to detect the composition of fecal gut microbiota in mice. In our study, PCoA analysis showed that the gut microbiota communities in mice were remarkably different between the three groups. At the phylum level, the DSS challenge induced abnormal changes in gut microbiota, such as a significant increase in the relative abundance of Actinobacteria and Proteobacteria (Cheng et al., 2022) and a significant decrease in the relative abundance of Bacteroidota compared with the CON group, however, the change caused by DSS cannot be relieved via Cramp treatment. Notably, Cramp treatment significantly elevated the abundance of Verrucomicrobiota compared to the DSS group. Verrucomicobiota has been reported to be one of the phyla present in the human gut, which has only one cultivated intestinal representative, *Akkermansia muciniphila* (Derrien et al., 2017). Studies have shown that the abundance of Verrucomicrobiota was significantly lower in UC patients with active phase, while significantly higher in the remission phases than that of the healthy control group at the phylum level (Zhu et al., 2022; Liu J. et al., 2023). Therefore, Verrucomicrobiota may offer potential prospects for future treatment of human UC. These results indicate that the Cramp treatment significantly altered the structure of gut microbiota.

Furthermore, at the genus level, DSS significantly reduced the relative abundance of the Lachnospiraceae_NK4A136_group and Alistipes in our study. The same as at the phylum level, cramp treatment did not improve the abundance of these genes. In addition, Cramp treatment increased the relative abundance of Parasutterella and Mucispirillum, while decreasing the relative abundance of Roseburia and Enterorhabdus, although DSS did not affect their abundance. Mucispirillum, a species reported to be protective against DSS-induced UC in mice (Nan et al., 2023) is identified as a marker of spontaneous colitis in mouse models of IBD (Vereecke et al., 2014). The increase in the relative abundance of Parasutterella following Cramp treatment in our study might relate to the potential role of Parasutterella in bile acid maintenance and cholesterol metabolism (Ju et al., 2019). In contrast to our findings, various studies reported a positive correlation between Roseburia abundance and IBD disease. However, in my results, Cramp treatment significantly reduced the abundance of Roseburia. This controversial result necessitates future investigation to elucidate the role of Roseburia in the IBD.

The gut microbiota can influence host health by educating and shaping host metabolism (Lavelle and Sokol, 2020). In the current study, changes in serum metabolome were explored by an untargeted metabolomics strategy to contribute to a better understanding of the mechanisms by which Cramp administration alters the gut microbiota of colitis. By combining univariate statistics analysis, we hypothesized that a total of 79 differential metabolites were identified between the CON and DSS groups. Notably, treatment of Cramp significantly improved eighteen metabolites when compared to the DSS group. Enrichment analysis indicated that these metabolites were involved in multiple metabolic pathways, including α-ainolenic acid metabolism, taurine and hypotaurine metabolism, sphingolipid metabolism, arachidonic acid metabolism, etc. All four metabolic pathways have been reported to be closely associated with inflammatory responses (Wang et al., 2019; Hadas et al., 2020; Linghang et al., 2020; Yuan et al., 2022). Notably, there was a significant correlation between the differential serum metabolome and gut microbiota.

## 4 Materials and methods

### 4.1 Animal experimental methods

Eight to ten weeks male C57BL/6 J mice were obtained from Jilin GENET-MED Biotechnology Co., Ltd. (Jilin, China). Mice were housed under standard laboratory conditions at constant temperature and humidity. After one week of acclimatization feeding, the mice were randomly divided into three groups: control (CON) group, DSS-treated (DSS) group, and DSS-treated group supplemented with 4 mg/kg/day Cramp (DSS + Cramp). The CON group was administered distilled water. The DSS and DSS + Cramp groups were administered 3% DSS (wt/vol; MW 36–50 kDa, MP Biomedicals, Solon, OH, United States) for 7 days. The CRAMP peptide was synthesized by ChinaPeptides Co., Ltd. (Shanghai, China) and injected intraperitoneally once daily starting on day 4 of the experimental period as described previously (Li et al., 2020; Fodor et al., 2022). Changes in body weight were measured daily during the experimental period. DAI was assessed as an average of the scores for the parameters mentioned, DAI = [weight loss (%) + stool consistency+rectal bleeding]/3 as previously described (Liu L. et al., 2023). At the end of the experimental, the colon and small intestine were carefully isolated and measured for length. The sample of colonic content was collected for gut microbiome analysis. The sample of blood was collected for biochemical assays and metabolomics analysis.

### 4.2 ELISA and oxidative stress markers assay

Mouse blood samples were placed at 4°C to precipitate serum, which was carefully aspirated after centrifuged at 2,500 rpm for 30 min.

Details of colon organ culture are described previously (Greten et al., 2004; Ismail et al., 2011). Briefly, the distal 2 cm of the colon was resected and recorded the wet weight. The colon tissue was longitudinally sliced open and rinsed with PBS containing penicillin/streptomycin, and further cut into 1 cm^2^ sections. Colon sections were incubated in RPMI 1640 media containing PS for 24 h, and cell-free supernatants were used to detect inflammatory cytokines.

Then, following the manufacturer’s instructions, the serum/colonic levels of interleukin-1β (IL-1β), IL-6, tumor necrosis factor-α (TNF-α), or monocyte chemotactic protein-1 (MCP-1) (BD Biosciences, San Diego, CA, United States) were measured using ELISA kit.

The levels of serum C-reactive protein (CRP) in serum were analyzed using a Mouse C-Reactive Protein ELISA kit (Beijing Solarbio Science & Technology Co., Ltd., Beijing, China) following the manufacturer’s instructions. The Malondialdehyde (MDA) content, superoxide dismutase (SOD) and Glutathione peroxidase (GSH-Px) activity in serum were detected following the kit instructions (Nanjing Jiancheng Bioengineering Institute, Jiangsu, China).

### 4.3 Histological assessment

To observe the histological changes, 4% paraformaldehyde-fixed colon and small intestine tissues were stained with hematoxylin and eosin (H&E). The morphological characteristics of the stained sections were examined using an Olympus CX23 microscope (Olympus, Tokyo, Japan). The calculation method for histological damage evaluation is as described previously (Ma et al., 2022).

### 4.4 Statistical analysis

Differences in quantitative data, expressed as mean ± SEM with statistical significance denoted by ^*^ *p* < 0.05, ^**^ *p* < 0.01, ^***^ *p* < 0.001. Group differences were assessed by Kruskal-Wallis test or ANOVA using Graph Pad Prism 7. Spearman’s correlation coefficients were computed for the relationships between differential serum metabolite levels and the relative abundances of gut microbiota at the genus and OTU levels, and a heatmap was designed for the correlation matrix using the Novogene Magic Cloud Platform.

The Supplementary materials include descriptions of additional methods.

## 5 Conclusion

In conclusion, our investigation demonstrated that Cramp treatment ameliorates DSS-induced colitis by decreasing the expression of inflammatory factors and improving intestinal barrier function. Meanwhile, our study revealed the alteration of the gut microbiome and serum metabolome by Cramp, suggesting a potential mechanism of Cramp in altering metabolism by modulating the gut microbiota.

## Data availability statement

The datasets presented in this study can be found in online repositories. The names of the repository/repositories and accession number(s) can be found at: NCBI—PRJNA1037342, EMBL-EBI—MTBLS8936.

## Ethics statement

The animal study was approved by Animal Care and Use Ethics Committee of Jilin Agricultural Science and Technology University. The study was conducted in accordance with the local legislation and institutional requirements.

## Author contributions

NJ: Conceptualization, Data curation, Methodology, Writing – original draft. ZL: Data curation, Methodology, Writing – original draft. HW: Investigation, Writing – original draft. LZ: Formal analysis, Writing – original draft. ML: Data curation, Writing – original draft. GL: Formal analysis, Writing – original draft. CL: Software, Writing – original draft. BW: Investigation, Writing – original draft. CZ: Project administration, Resources, Writing – review & editing. LL: Funding acquisition, Supervision, Writing – review & editing.
