# Supplementary material for: Alterations in metabolome and microbiome: new clues on cathelicidin-related antimicrobial peptide alleviates acute ulcerative colitis
Source: Front Microbiol. 2024 Feb 6;15:1306068. doi: 10.3389/fmicb.2024.1306068 (PMC10877057; doi:10.3389/fmicb.2024.1306068)
Supplement: Supplementary file 1 [file Table_1.pdf]

Supplementary Material

Alterations in Metabolome and Microbiome: New Clues on Cathelicidin-Related Antimicrobial Peptide Alleviates Acute Ulcerative Colitis

Nan Jiang *et al.*

Material and Methods

Immunohistochemistry

After one hour block at room temperature, colon tissue sections were specifically conjugated to target proteins using a primary antibody anti-E-Cadherin (Cell Signaling Technologies, Beverly, MA, USA) overnight at 4℃, followed by incubation with HRP-conjugated secondary antibodies at 4℃ for 1 h. Signal transitions were detected using the DAB substrate kit (DAKO, Carpinteria, CA, USA).

RNA extraction and Real-Time Quantitative PCR (RT-qPCR)

Total RNA was extracted from a segment of the colon using RNAiso Plus (Takara Bio Inc, Kusatsu, Shiga, Japan) and purified using lithium chloride (LiCl) as previously described (Viennois et al., 2013). Then the RNA was reverse-transcribed into cDNA using a PrimeScriptTM RT reagent Kit with gDNA Eraser (Perfect Real Time) (Takara Bio Inc, Kusatsu, Shiga, Japan). Quantitative PCR (qPCR) was performed with gene-specific primers (Table S2) on an ABI StepOnePlus real-time PCR thermocycler (Thermo Fisher Scientific, Waltham, MA, USA) with TB GreenPremix Ex Taq (Tli RNaseH Plus) (Takara Bio Inc, Kusatsu, Shiga, Japan). The data were normalized to the 18s level for mRNA. Relative changes in mRNA expression were calculated using the $^{\Delta\Delta}CT$ method.

Myeloperoxidase (MPO) Activity

Colonic tissue homogenates were used to measure MPO activity. MPO activity was evaluated using a fluorescence-based activity assay kit (Abcam, Cambridge, MA, USA), according to the manufacturer's instructions, to calculate the MPO concentration in each sample.

Metabolite extraction

A 50 μl of serum samples were added with 3 times methanol for protein precipitation. Then the resulting mixture was centrifuged at 12,000g for 15min at 4℃, and the supernatant was lyophilized using a freeze dryer. The lyophilized samples were re-

dissolved in 200ul methanol/water (1:1), and centrifuged at 15,000g for 15min at 4℃. Mixed quality control (QC) samples were made by mixing equal volumes of each sample, and method validation was carried out using the QC samples.

LC-MS analysis of serum metabolite

Serum sample extracts were analyzed using an LC-MS analysis platform (Ultimate 3000LC, Q-Orbitrap, Thermo Fisher Scientific, San Jose, CA, USA) and a Hyperisil Gold (3μm, 100×2.1) column (Thermo Fisher Scientific, San Jose, CA, USA). The column temperature was 40℃ and the injection volume was 5μl. A formic acid aqueous solution (0.1%, Phase A) and 0.1% formic acid acetonitrile solution (0.1%, Phase B) at a flow rate of 0.4ml/min under the following gradient program: 3%B (0-1min); 3-70%B (1-8min); 70%B (8-10min); 70-90% (10-17min); 90-100% (17-18min); 100% (18-21min); 100-3%B (21-23min); 3%B (23-26min).

Mass spectrometry was operated in both positive and negative ion modes. Profile data was acquired in the 100-1200m/z range. Tandem MS information was obtained under DDMS2 (TOP 3) mode. The acquisition was performed at a resolution of 17,500, with the ramped normalized collision energy of 30, 50, and 60. The key parameters of the ion source were set as follows: The capillary voltage is 3.5 Kv in positive ion mode or -3.2 Kv in negative ion mode, sheath gas flow 50 arb, auxiliary gas flow 15 arb, sweep gas flow 2 arb, capillary temperature 350 °C. Before sample analysis, the mass spectrometer was calibrated using Pierce™ calibration solution provided by Thermo Scientific (Thermo Fisher Scientific, San Jose CA, USA). All samples were kept at 4 °C during analysis.

Data processing and metabolites identification

The raw LC-MS data was processed using MS-Dial software 4.90 for peak extraction, alignment, and normalization before being exported as a dataset including the sample code, peak label, and peak intensity. The metabolite identification of MS1 and MS2 data was also performed by searching the data obtained from the database provided by MS-Dial software.

Gut microbiome analysis

Mice feces were collected and stored at -80 ℃. The feces samples were sent to Novogene Co., Ltd. (Beijing, China) for 16S rRNA sequencing under dry ice preservation. Briefly, total genome DNA from samples was extracted using CTAB method. According to the concentration, DNA was diluted to 1ng/μL using sterile water. The primers 341F and 806R (Table S2) were selected to amplify the V3-V4 region of the 16S rRNA gene. DNA libraries were constructed using the TruSeq® DNA PCR-free Sample Preparation Kit, and library was sequenced on an Illumina NovaSeq platform. Paired-end reads were merged using FLASH (V1.2.7,

http://ccb.jhu.edu/software/FLASH/) [1]. Quality filtering on the raw tags were performed according to the QIIME (V1.9.1, http://qiime.org/scripts/split_libraries_fastq.html) quality controlled process [2]. Sequences analysis were performed by Uparse software (Uparse v7.0.1001, http://drive5.com/uparse/) [3]. Additionally, dilution curves, relative abundance of species, principal coordinate analysis (PCoA) and LDA effect size (LEfSE) analysis were performed in R software (Version 2.15.3). Data were analyzed using the Novogene Magic Cloud Platform (https://magic.novogene.com).

Western blot analysis

Colon tissues were homogenated and lysed in ice-cold lysis buffer (Beyotime Biotechnology, Shanghai, China) supplemented with the Protease and a phosphatase inhibitor cocktail (Beyotime Biotechnology, Shanghai, China). The supernatant was then collected after a 15 min centrifugation at 12,000 rpm, 4 ℃. The protein concentration was determined using the BCA protein assay reagent kit (TermoFisher Scientific, Rockford, USA). Thirty micrograms of protein were loaded onto SDS-PAGE gels (EpiZyme Biotechnology, Shanghai, China), and the separated proteins were transferred to PVDF members (Millipore, Billerica, MA, USA). The membranes were incubated with primary antibodies: anti-Claudin 1, anti-Occludin, anti-ZO-1, and anti-β-actin (Cell Signaling Technologies, Beverly, MA). The secondary antibody was purchased from Cell Signaling Technologies (Cell Signaling Technologies, Beverly, MA). Images were obtained by the Syngene G: box (Gene Company Limited, Hongkong, China) using ECL Western blot detection reagent (Pierce) (TermoFisher Scientific, Rockford, USA).

Biomarker screening

MetaboAnalyst 5.0 service performed a pathway enrichment analysis. Interquartile range (IQR) is used to filter data, normalization by sum pairs for normalization, pareto scaling is used to scale the data, and then the multi-factor analysis is performed. Principal component analysis (PCA) and orthogonal partial least squares discriminant analysis (OPLS-DA) were then used to identify differentiated metabolites between the two groups.

Supplementary Table 1. Identified Metabolites and Their Changes in Serum from Different Groups

No.	Metabolite name	HMDB ID	VIP	Rt (min)	exact mass	DSS/CON	DSS+Cramp/DSS
1	Sphinganine	HMDB0000269	1.51	15.77	324.28632	↑**	↓
2	Dihomo-gamma-linolenic acid	HMDB0002925	1.16	14.71	307.26111	↑*	↓
3	Hyodeoxycholic acid	HMDB0000733	1.76	13.30	391.28467	↓***	↓#
4	Lithocholic acid	HMDB0000761	1.57	13.18	375.2887	↑**	↓
5	12-Hydroxystearic acid	HMDB0061706	1.32	12.16	299.25778	↑*	↑
6	12-KETE	HMDB0013633	1.14	11.69	317.21063	↑*	↓
7	Alpha-dimorphecolic acid	HMDB0004670	1.29	11.67	319.22351	↑*	↓
8	Isopalmitic acid	HMDB0031068	1.37	11.04	295.22418	↑*	↓
9	2-Hydroxyhexadecanoic acid	HMDB0031057	1.48	10.74	271.22644	↑**	↓
10	LysoPC(18:0)	HMDB0010384	1.19	10.34	546.35071	↑*	↑
11	Alpha-Linolenic acid	HMDB0001388	1.54	10.28	279.23007	↑**	↓
12	12-HEPE	HMDB0010202	1.39	10.01	317.21057	↑*	↓
13	Urapidil	HMDB0259712	1.43	8.94	386.2254	↓*	↓
14	Sphinganine 1-phosphate	HMDB0001383	1.19	8.84	382.26956	↑*	↓
15	9,10-DHOME	HMDB0004704	1.62	8.83	313.23773	↑**	↓
16	12,13-DHOME	HMDB0004705	1.35	8.60	313.23749	↑*	↓
17	15(S)-HPETE	HMDB0004244	1.57	8.55	335.22205	↑**	↓##
18	Prostaglandin E1	HMDB0001442	1.62	8.10	353.23157	↑**	↓
19	(-)-Arctigenin	HMDB0030087	1.43	8.03	371.20566	↓*	↓
20	Isotaxiresinol	HMDB0253695	1.47	8.03	345.15393	↓*	↓
21	3-Epiaphidicolin	HMDB0245864	1.29	8.00	337.23672	↑*	↓
22	Kaempferol	HMDB0005801	1.89	7.89	285.20581	↑***	↓###
23	8,15-DiHETE	HMDB0010219	1.25	7.80	335.22089	↑*	↓
24	Sebacic acid	HMDB0000792	1.42	7.65	225.11041	↑***	↓##
25	N-Octanoyl-L-homoserine lactone	HMDB0255213	1.40	7.32	228.15768	↓*	↓
26	Sphingosine	HMDB0000252	1.34	6.93	300.28696	↑*	↓
27	Indole-3-propionic acid	HMDB0002302	1.52	6.60	188.07007	↑**	↓#
28	5alpha-Cholestanol	HMDB0000908	1.64	5.85	387.23706	↑**	↓
29	Dihydroferulic acid	HMDB0062121	1.41	5.22	195.06488	↑**	↓
30	Normetanephrine	HMDB0000819	1.23	5.15	182.08078	↑*	↓#

31	Kynurenic acid	HMDB0000715	1.52	5.07	190.04825	↑***	↓##
32	Thiazolidine-4-carboxylic acid	HMDB0258979	1.63	4.89	134.02594	↓**	↓
33	1H-Benzotriazole	HMDB0244873	1.66	4.82	120.05496	↓***	↑
34	Acetophenazine	HMDB0015196	1.32	4.81	412.20746	↑*	↓
35	Nifekalant	HMDB0255595	1.25	4.67	404.19009	↓*	↓
36	N-Acetylneuraminate	HMDB0341204	1.57	4.66	308.09775	↓**	↑
37	Mycophenolic acid	HMDB0015159	1.47	4.63	319.13895	↓*	↓
38	Acetaminophen glucuronide	HMDB0010316	1.31	4.30	326.08774	↑*	↓#
39	Digitoxin	HMDB0015468	1.32	4.07	763.36096	↑*	↓
40	Gentiopicroside	HMDB0252696	1.53	3.67	401.10773	↑**	↓#
41	Isobutyrylglycine	HMDB0000730	1.26	3.42	144.06487	↑*	↓#
42	4-Acetamidobutanoic acid	HMDB0003681	1.11	3.41	146.08	↑*	↓#
43	N-Methyl-L-isoleucine	HMDB0341355	1.68	3.32	146.1165	↑***	↓
44	Scopolamine	HMDB0003573	1.40	3.27	304.17249	↓**	↓
45	gamma-Glutamylmethionine	HMDB0034367	1.59	3.14	277.08524	↓**	↑
46	Ascorbic acid	HMDB0000044	1.28	2.33	175.02296	↓*	↑
47	Allopurinol	HMDB0014581	1.81	2.24	135.02888	↓***	↑
48	Orotidylic acid	HMDB0000218	1.73	2.24	366.99728	↓***	↑
49	Inosine	HMDB0000195	1.70	2.20	269.08572	↓***	↑
50	Methylmalonic acid	HMDB0000202	1.71	2.19	119.03448	↓***	↑
51	D-Ribose 5-phosphate	HMDB0001548	1.90	2.18	229.01056	↓***	↑
52	Imidazoleacetic acid	HMDB0002024	1.52	2.14	127.04919	↑***	↓#
53	Guanosine	HMDB0000133	1.50	2.13	284.09662	↓***	↑
54	Guanine	HMDB0000132	1.70	2.08	152.0555	↓***	↑
55	Guanosine monophosphate	HMDB0001397	1.18	1.72	362.05048	↓*	↓
56	Hypoxanthine	HMDB0000157	1.60	1.64	137.04501	↓**	↑
57	Uridine	HMDB0000296	1.60	1.56	243.0609	↓**	↑
58	Prolylproline	HMDB0011180	1.23	1.49	213.12192	↑*	↓
59	Caffeine	HMDB0001847	1.42	1.18	195.00153	↑**	↓
60	lumichrome	HMDB0254199	1.35	1.18	243.02457	↑**	↓#
61	Cyclophosphamide	HMDB0014672	1.45	1.18	283.01648	↑***	↓##
62	Fructose 6-phosphate	HMDB0000124	1.30	1.16	259.02148	↑*	↓##
63	Norvaline	HMDB0013716	1.41	1.06	140.06718	↓**	↑

64	Hypotaurine	HMDB0000965	1.30	1.01	110.03428	↓*	↓
65	Beta-Carboline	HMDB0003070	1.23	0.99	192.08496	↑*	↓#
66	Gulonic acid	HMDB0003290	1.18	0.99	177.03874	↓*	↑
67	p-Aminobenzoic acid	HMDB0001392	1.51	0.99	138.05392	↓*	↓
68	Beta-Carboline	HMDB0012897	1.70	0.98	186.10439	↓***	↓##
69	Ethyl butylacetylaminopropionic acid	HMDB0246904	1.48	0.89	216.15749	↑***	↑
70	4-Guanidinobutanoic acid	HMDB0003464	1.62	0.86	146.09134	↑***	↓#
71	Guaiazulene	HMDB0036648	1.62	0.86	199.14241	↑***	↑
72	Ranitidine-S-oxide	HMDB0257111	1.26	0.76	331.14487	↑**	↓
73	2-Aminobenzothiazole	HMDB0245009	1.17	0.64	151.0341	↑*	↓
74	Allantoin	HMDB0000462	1.37	0.63	176.07556	↑*	↓
75	AMPA	HMDB0240707	1.20	0.63	112.00567	↑*	↓
76	Chinizarin	HMDB0257042	1.25	0.63	263.02631	↑*	↓
77	Ethyl glucuronide	HMDB0010325	1.30	0.63	261.02829	↑*	↓
78	1-hydroxymidazolam	HMDB0061089	1.58	0.62	342.08636	↑**	↓
79	Glucuronolactone	HMDB0242712	1.46	0.62	177.04124	↑**	↓

$^{*}P < 0.05$, $^{**}P < 0.01$, $^{***}P < 0.001$ DSS *vs* CON. $^{\#}P < 0.05$, $^{\#\#}P < 0.01$ DSS+Cramp *vs* DSS group.

Supplementary Table 2: Primer Sequences for q-PCR analysis

Gene	Sequence (Forward/Reverse 5' to 3')	
IL-6	ATGAAGTTCCTCTCTGCAAGAGAC	CACTAGGTTTGCCGAGTAGATCTC
MCP-1	GGCTCAGCCAGATGCAGT	GAGCTTGGTGACAAAAACTACAG
TNF-α	CACCACCATCAAGGACTCAA	AGGCAACCTGACCACTCTCC
18s	CTAACCCGTTGAACCCCATT	CCATCCAATCGGTAGTAGCG
341F	CCTAYGGGRBGCASCAG	
806R	GGACTACNNGGGTATCTAAT	

References

1. Magoc, T. and S.L. Salzberg, FLASH: fast length adjustment of short reads to improve genome assemblies. Bioinformatics, 2011. **27**(21): p. 2957-63.
2. Caporaso, J.G., et al., QIIME allows analysis of high-throughput community sequencing data. Nat Methods, 2010. **7**(5): p. 335-6.
3. Edgar, R.C., UPARSE: highly accurate OTU sequences from microbial amplicon reads. Nat Methods, 2013. **10**(10): p. 996-8.
